# Acute Blindness in the Elderly With Craniopharyngioma

**DOI:** 10.7759/cureus.26880

**Published:** 2022-07-15

**Authors:** Ng Kwang Sheng, Wan-Hazabbah Wan Hitam

**Affiliations:** 1 Department of Ophthalmology and Visual Science, School of Medical Sciences, Universiti Sains Malaysia, Kubang Kerian, MYS; 2 Ophthalmology Clinic, Hospital Universiti Sains Malaysia, Universiti Sains Malaysia, Kubang Kerian, MYS

**Keywords:** surgery, acute, blindness, elderly, craniopharyngioma

## Abstract

The afferent visual system is one of the most common structures involved in patients with craniopharyngioma, and the manifestations include deficits in visual acuity, color vision, and visual fields. Here, we report a case of craniopharyngioma that presented with acute blindness in an elderly man. A healthy 54-year-old man presented with an acute progressive blurring of vision and became blind in six weeks. He developed symptoms of increased intracranial pressure only a week after becoming blind. On examination, visual acuity in both eyes was no perception of light (NPL). He also had left esotropia with restriction of left eye abduction. Both pupils were not responsive to light. The anterior segment was normal in both eyes. Fundoscopy showed bilateral pale optic discs. Computed tomography scan and magnetic resonance imaging revealed a suprasellar mass consistent with craniopharyngioma that compressed the optic chiasma and adjacent brain structures with the presence of hydrocephalus. He underwent uneventful tumor debulking surgery. However, his vision remained NPL postoperatively. Ocular manifestations could be the only symptoms in craniopharyngioma. The delayed presentation may lead to a guarded prognosis.

## Introduction

Craniopharyngiomas are generally benign in nature [1]. However, these tumors are aggressive locally toward the sellar and suprasellar areas, which are adjacent to some important neural and vascular structures [2]. Ocular manifestations such as progressive visual deterioration are common in these patients; however, sudden vision loss is rare [3]. On the other hand, complete visual recovery of total blindness after surgical decompression has been reported in patients diagnosed with craniopharyngioma [4]. Here, we report a case of craniopharyngioma in an elderly man who presented with acute blindness.

Part of this article was previously presented as a poster at the 35th Singapore-Malaysia Joint Meeting in Ophthalmology from January 17 to 19, 2020, in Singapore.

## Case presentation

**Figure 1:**
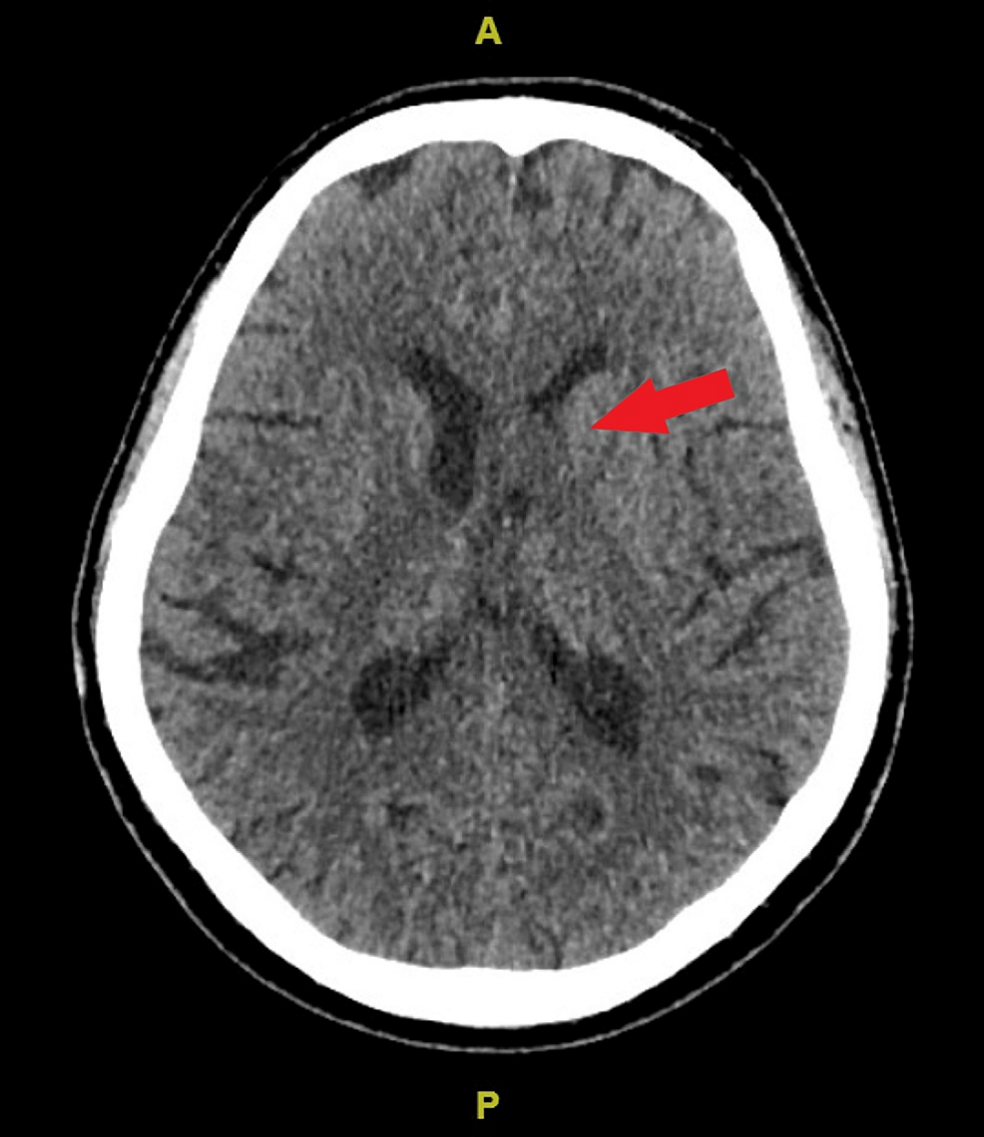
Axial cut of the plain computed tomography image showed a mass compressed on the left lateral ventricle (red arrow).

**Figure 2:**
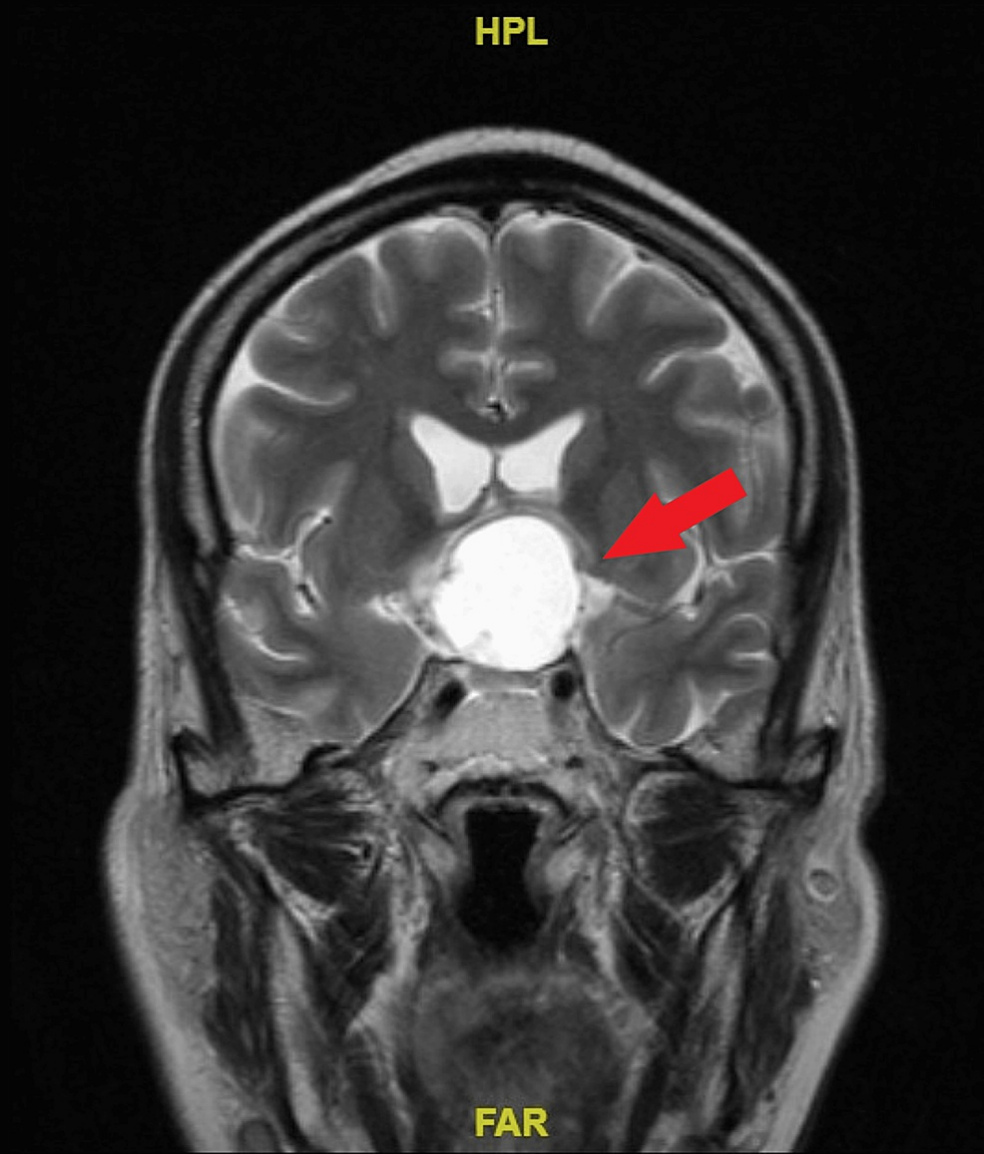
Coronal cut of the T2-weighted magnetic resonance imaging showed a well-defined hyperintense mass at the suprasellar area compressing the adjacent brain structure (red arrow).

A healthy 54-year-old man presented with an acute progressive blurring of vision in both eyes. It started with generalized blurring and he became totally blind within six weeks. He experienced horizontal diplopia before total blindness. Otherwise, there was no eye pain, eye redness, or proptosis. He started to develop headaches only one week after becoming blind. The headache was described as frontal, throbbing in nature, and aggravated on straining. However, there was no nausea or vomiting. He also had lethargy, loss of appetite, and loss of weight. His ocular history was unremarkable. On examination, the visual acuity in both eyes was no perception of light (NPL) in all four quadrants. The anterior segment in both eyes was normal. Bilateral pupils were dilated 5 mm in diameter and not responsive to light. The intraocular pressure (ICP) was normal in both eyes. There was left esotropia on the primary gaze with restriction in abduction. Fundoscopy examination revealed a pale optic disc in both eyes. Computed tomography (CT) scan and magnetic resonance imaging (MRI) revealed a suprasellar mass consistent with craniopharyngioma that compressed the optic chiasma and adjacent brain structures with the presence of hydrocephalus (Figures 1, 2). He underwent uneventful tumor debulking surgery. Postoperatively, the patient was stable. However, his visual acuity of both eyes remained NPL.

## Discussion

Craniopharyngiomas are rare tumors, with the overall incidence ranging from 0.12 to 0.13 per 100,000 per year [5]. They comprise about 2-5% of primary intracranial tumors [6]. They may occur at any age but have two peaks in children aged 5-14 and adults aged 50-74 [5], as seen in our case. They are epithelial tumors that develop from the craniopharyngeal duct; however, their pathogenesis is not known [6]. Regarding pathological features, the majority are mixed solid and cystic tumors, with only some completely solid or cystic [7]. The molecular pathogenesis of craniopharyngioma is not well-understood yet [8]. Mutations of β-catenin, which plays an important role in the Wnt signaling pathway, are believed to be responsible for the development of this tumor [9,10].

As for the clinical features, they are dependent on the size, location, and growth of the tumor, as well as the relationship with the surrounding structures. The most common presentation is visual disturbance, and studies have shown that the prevalence of visual disturbance could be as high as 65-82% [3,11,12]. Sudden visual loss is a rare presentation. However, there are case reports of patients who presented with acute blindness and had visual recovery post-decompressive surgery [4,13]. Unfortunately, our patient did not regain his vision after surgery. This could be due to much more severe damage to the optic nerves for a longer duration before initiation of treatment, which was about two months compared to three and ten days in the other cases reported with visual recovery. Visual field defect is a very common symptom with a prevalence of 86%. Bitemporal hemianopia is the most common visual field deficit [11]. Our patient had total blindness upon presentation and visual field examination was not feasible. Besides, he denied experiencing any visual field defect. The mechanism of visual disturbances is due to the mass effect of the tumor by compressing and stretching the optic nerves, which could lead to irreversible damage once secondary ischemic insult has developed [4]. Optic atrophy, as in our case, is a common finding according to reported studies, accounting for 21% of the prevalence [12].

On the other hand, symptoms of increased ICP are very common and have been reported in 75% of cases. However, cranial nerve palsy is not as common with an 11% prevalence in the same study [12]. Our patient experienced symptoms of increased ICP only one week after becoming blind. Other symptoms such as lethargy, sleeping disturbances, weight gain or loss, impaired sexual function, and menstrual disorders could be due to hormonal disturbances [1].

Regarding diagnosis, any suspicion should be based on clinical and radiological findings. CT and/or MRI are the choices for radiological investigation. CT has better visualization of calcifications and bony structures, whereas the assessment of tumor morphology and relationship with surrounding structures is better achieved by MRI. Therefore, MRI is the imaging of choice in preparation for surgery [14]. Hydrocephalus is a common finding in craniopharyngioma, accounting for 20% of reported cases [15].

Surgery remains the first-line management. Complete resection is the goal of surgical treatment. Surgical complications might occur if the tumor size is too big or if the tumor is in close relation to other vital structures. Partial resection would be favorable if complete resection is not possible. Radiotherapy is the treatment of choice if the patient is not suitable for surgery, in cases with incomplete tumor resection, or in recurrent cases. Chemotherapy is another treatment option, especially for cystic tumors [1]. Visual recovery is possible following surgery. A previous study showed improvement in visual field defect (36%) and visual acuity (30%) after treatment [16]. The rate of recurrence of craniopharyngioma postoperatively is high and has been reported to be 24.5% in a study of 106 patients [17].

## Conclusions

Ocular manifestations of rapid progression can develop much earlier than other symptoms in craniopharyngioma. Differential diagnosis of any suspicious ocular feature should not be limited to just ocular in origin as it could be the only or earlier symptom or sign. A high index of suspicion and timely imaging can be vision or lifesaving.
